# IL-6 and Mouse Oocyte Spindle

**DOI:** 10.1371/journal.pone.0035535

**Published:** 2012-04-20

**Authors:** Jashoman Banerjee, Rakesh Sharma, Ashok Agarwal, Dhiman Maitra, Michael P. Diamond, Husam M. Abu-Soud

**Affiliations:** 1 Department of Obstetrics and Gynecology, Wayne State University, Detroit, Michigan, United States of America; 2 Center for Reproductive Medicine, Glickman Urological and Kidney Institute, Cleveland Clinic, Cleveland, Ohio, United States of America; 3 Department of Biochemistry and Molecular Biology, Wayne State University School of Medicine, Detroit, Michigan, United States of America; Institut Jacques Monod, France

## Abstract

Interleukin 6 (IL-6) is considered a major indicator of the acute-phase inflammatory response. Endometriosis and pelvic inflammation, diseases that manifest elevated levels of IL-6, are commonly associated with higher infertility. However, the mechanistic link between elevated levels of IL-6 and poor oocyte quality is still unclear. In this work, we explored the direct role of this cytokine as a possible mediator for impaired oocyte spindle and chromosomal structure, which is a critical hurdle in the management of infertility. Metaphase-II mouse oocytes were exposed to recombinant mouse IL-6 (50, 100 and 200 ng/mL) for 30 minutes and subjected to indirect immunofluorescent staining to identify alterations in the microtubule and chromosomal alignment compared to untreated controls. The deterioration in microtubule and chromosomal alignment were evaluated utilizing both fluorescence and confocal microscopy, and were quantitated with a previously reported scoring system. Our results showed that IL-6 caused a dose-dependent deterioration in microtubule and chromosomal alignment in the treated oocytes as compared to the untreated group. Indeed, IL-6 at a concentration as low as 50 ng/mL caused deterioration in the spindle structure in 60% of the oocytes, which increased significantly (P<0.0001) as IL-6 concentration was increased. In conclusion, elevated levels of IL-6 associated with endometriosis and pelvic inflammation may reduce the fertilizing capacity of human oocyte through a mechanism that involves impairment of the microtubule and chromosomal structure.

## Introduction

Interleukin-6 (IL-6) is a pleotropic cytokine which is known to activate acute phase proteins and maintain chronic inflammatory states ranging from cardiovascular diseases to infertility [1]. For example, IL-6 has been known to be involved in the pathogenesis of small vessel diseases, accelerate joint inflammation and destruction in rheumatoid arthritis, and also contribute to neurovascular disease such as dementia [2], [3], [4]. This pre-inflammatory cytokine is known to be expressed and reactive oxygen species (ROS) are known to be elevated in patients with chronic pain syndromes, acute pelvic inflammatory disease, idiopathic infertility and patients with endometriosis [5], [6], [7], [8], [9].

In oocytes, as in other cells, ROS play an important role as regulatory mediators of intracellular signaling responsible for cellular functions in biological systems [10]. In contrast, under pathological conditions, substantially elevated ROS are hazardous, resulting in mutations, inactivation or loss of mitochondrial DNA, and synthesis and accumulation of oxidized proteins. Similarly, oxidative stress is known to affect the membrane lipid composition, decrease the concentrations of antioxidants, and increase cytosolic Ca^2+^ which hampers cellular function [11], [12], [13], [14]. Recently, we have demonstrated that oocytes exposed to increased concentration of ROS such as superoxide (O_2_^•−^), hydrogen peroxide (H_2_O_2_), and hypochlorous acid (HOCl) undergo deterioration of quality as assessed by an increase in zona pellucida dissolution time, increase in ooplasmic microtubule dynamics, and major loss in the cortical granule status, all of which are major markers of oocyte aging phenomena [15]. These findings may explain high reproductive failure known to occur with advancing age, obesity, diabetes mellitus, and a myriad of other clinical conditions [16], [17], [18], [19]. ROS are known to be one of the major stimuli of cytokines which may mediate oxidative stress, and potentially alter the redox equilibrium in various cell types [20], [21], [22]. IL-6 has been correlated with ROS in infertile males with varicocele and has been shown to contribute to male infertility [23]. In this work, we explored the direct effect of various concentrations of IL-6 on the metaphase-II mouse oocyte spindle and chromosomal alignment *in vitro*, as a possible mechanism of deterioration of oocyte quality in patients with pelvic inflammatory states where IL-6 levels are elevated.

## Results

### Effect of IL-6 on microtubules

**Figure 1:**
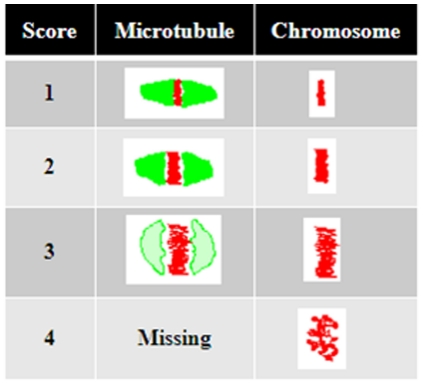
Algorithm of scoring alterations in microtubule and chromosomal alignment on the Metaphase-II mouse oocyte based on previously published data.

**Figure 2:**
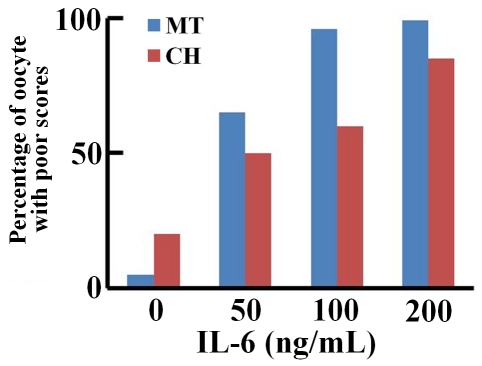
Percentage of oocytes with “Poor” scores (score 3, 4) in microtubule (MT) and chromosomal alignment (CH) at different concentrations of IL-6.

To determine the effects of IL-6 on metaphase-II mouse oocytes, oocytes were exposed to recombinant mouse IL-6 (50, 100 and 200 ng/mL) for 30 minutes and subjected to indirect immunofluorescent staining to identify alterations in the microtubule and chromosomal alignment compared to untreated controls. Scores 1 and 2 were combined as “Good” score and 3 and 4 as “Poor” score (Figure 1, see *Materials and methods* section for more details). The majority of oocytes (86.4%) had “Good” microtubular scores defined as those with scores 1 and 2, in the unexposed controls compared to only 9.5% in the group exposed to 200 ng/mL of IL-6 (p<0.01) (Figure 2, Table 1). Alterations in the microtubule scores were seen in IL- 6 concentrations as low as 50 ng/ml. At an IL-6 concentration of 50 ng/ml 36% of oocytes had “Good” and 64% had “Poor” scores. At 100 ng/mL, 23.5% of oocytes demonstrated “Good” scores whereas more than 75% had “Poor” scores. Most of the oocytes (90.5%) demonstrated poor scores when treated with 200 ng/ml of IL-6. Thus IL-6 caused a dose dependant deterioration of oocyte microtubules.

**Table 1:** Percentage of oocytes with “Good” (score 1, 2) and “Poor” (score 3, 4) score in microtubular (MT) and chromosomal (CH) alignment at different concentrations of IL-6.

		Percentage of oocytes with “Good” and “Poor” outcomes		
IL- 6 (ng/mL)	Oocyte component	Score 1 & 2 (Good)	Score 3 & 4 (Poor)	p- value
0	Microtubule	86. 4%	13. 6%	<0.01
	Chromosome	89. 8%	10. 2%	
50	Microtubule	36. 0%	64. 0%	<0.01
	Chromosome	68. 0%	32. 0%	
100	Microtubule	23. 5%	76. 5%	<0.01
	Chromosome	23. 5%	76. 5%	
200	Microtubule	9. 5%	90. 5%	<0.01
	Chromosome	14. 3%	85. 7%	

### Effect of IL-6 on chromosomal alignment

Similar to the effect observed in the microtubules, overall comparison between untreated and treated groups demonstrated a trend of increased “Poor” scores with increasing IL-6 concentrations in chromosomal alignment. Oocytes exposed to 200 ng/mL of IL-6, showed higher “Poor” scores (85.7%) compared to 10.2% in the unexposed control group (p<0.01). At 50 ng/mL 32% had “Poor” scores compared to 68% with “Good” scores in chromosomal alignment (Table 1; Figure 2).

**Figure 3:**
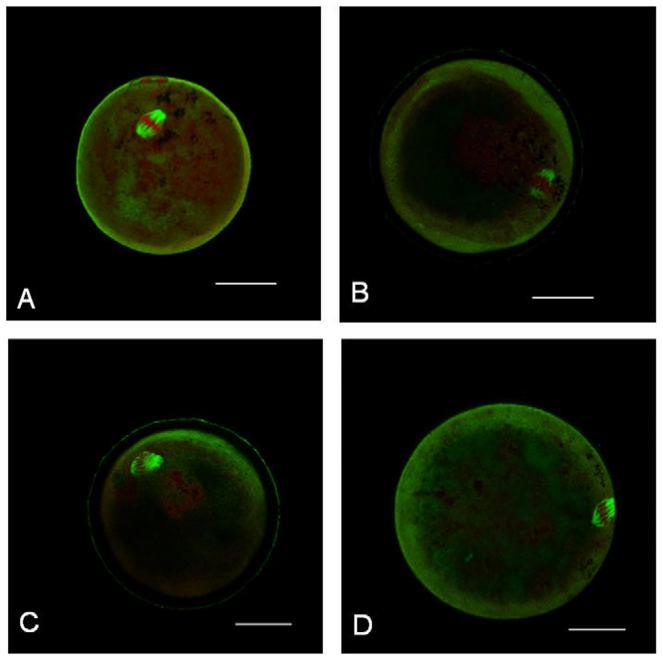
Confocal images of oocytes. **A**. Unexposed control; **B**. Effect of IL-6 at 50 ng/mL; **C**. Effect of IL-6 at 100 ng/mL; and **D**. Effect of IL-6 at 200 ng/mL Scale – 1 pixel, 5 mm.

Our findings represent a concentration dependant alteration of the metaphase-II oocyte microtubule and chromosomal alignment when exposed to recombinant mouse IL-6. An IL-6 concentration greater than 50 ng/mL resulted in an increase in “Poor” scores in both the microtubules and chromosomes. Confocal images demonstrating alterations in microtubules and chromosomal alignment at different concentrations are shown in (Figure 3A–D).

## Discussion

Our current finding, for the first time, demonstrated a direct link between increasing concentrations of IL-6 and the deterioration in morphology of the microtubule and chromosomal alignment in metaphase-II mouse oocytes. This alone may be important in biologic settings such as pelvic inflammation and endometriosis where IL-6 levels are elevated.

It is well established that maintenance of the integrity of the mature oocyte spindle is vital for proper cell division and embryo formation [24]. Indeed, abnormal spindle dynamics mediated by IL-6 elevation is known to lead to aneuploidy, failure of fertilization, early loss of pregnancy [19], or low reproductive outcome under certain pathological conditions such as in patients with endometriosis and pelvic inflammation [18]. It is unclear what specifically induces these alterations in the oocytes, but inducers such as TNF- α, and ROS associated with oxidative stress have been shown to alter the oocyte morphology and spindle dynamics contributing to infertility [15], [25], [26]. Thus, the effect of IL-6 on oocyte quality could be explained by its ability to directly affect the metaphase-II oocyte through its negative effect on the spindle structure and chromosomal alignment or indirectly by involvement of reactive oxygen species.

Oxidative stress is well known to increase IL-6 production in several types of cells. As for example, oxidized low density lipoprotein can upregulate nuclear factor kappa B (NF-Kb) mediated IL-6 generation in endothelial cells [27], and acute hypoxia leading to generation of oxidative stress have been documented to increase IL-6 production in hepatocytes [28]. It has also been documented that the addition of ROS scavengers resulted in the inhibition of the IL-6 response to ROS stimulation, suggesting that ROS may be, at least in part, involved in IL-6 production [29], [30], [31]. Previously, we have shown that ROS such as superoxide (O_2_^•−^), hypochlorous acid (HOCl), and hydrogen peroxide (H_2_O_2_) can alter the oocyte quality manifested by hypergranulated cytoplasm, absence of perivitelline space, abnormal spindle dynamics [15]. More recently, we have demonstrated that HOCl induced alteration in the microtubule and chromosomal structure of metaphase-II mouse oocytes *in vitro*, a process that can be inhibited by melatonin (article in press) [32]. Choi *et al.* demonstrated that H_2_O_2_ at 12.5 nmol/mL initiated the deterioration in the microtubule and chromosomal structure whereas [26] HOCl at 50 nmol/mL demonstrated similar effects (article in press) [32]. Our current study demonstrated similar changes assessed by the same scoring technique for the alterations of the spindle and chromosomal structure at concentration of 50 ng/mL of IL-6. Hence, it is evident that pathological concentrations of ROS and cytokines whose generation in biological systems is interdependent in inflammatory states can affect the oocyte spindle structure. Thus deterioration of the oocyte spindle may be a direct effect of ROS generated from inflammation or hypoxia or secondary to cytokines like IL-6 augmented by ROS generation.

Various authors have shown that women with endometriosis have higher peritoneal fluid concentrations of IL-6 compared to those who are normal [33], [34], [35], [36]. Peritoneal fluid macrophages are known to express IL-6 and soluble IL-6 receptors through which this cytokine is known to exert its effects in pelvic inflammatory states [37]. A recent animal study demonstrated that IL-6 can increase the rate of meiotic arrests and germinal vesicle breakdown in bovine species in inflammatory states induced by treatment with lipopolysaccharide [38]. Another group has demonstrated that peritoneal fluid obtained from patients with endometriosis affects oocyte microtubule and chromosomal structure leading to altered oocyte quality [25] which could be prevented by treatment with L-carnitine [39]. L-carnitine is known to down-regulate cytokines such as IL-1, IL-6, and TNF-α and increase their clearance in rats implanted subcutaneously with sarcoma tumor [40]. Since the metaphase-II oocyte gets exposed to the higher peritoneal fluid concentrations of IL-6 after ovulation in patients with inflammatory disorders, it may undergo deterioration of spindle structure and chromosomal alignment contributing to failure of fertilization and poor reproductive outcomes.

Other than peritoneal fluid, follicular environment in endometriosis demonstrates higher IL-6 which may hamper oocyte quality prior to ovulation [41], [42]. Interestingly, IL-6 has been shown to play a role in regulating mouse cumulus cell expansion as a physiological process in the ovarian follicle. This study also indicated that the effects of IL-6 on cumulus cells and the oocyte, may be detrimental if elevated within the follicle at inappropriate or for extended periods of time, such as during chronic infections, endometriosis and in obese patients. Therefore, impaired fertility associated with these conditions could be related to the direct effects of abnormally high levels of IL-6 and other potent cytokines that can impair ovarian follicular cell function and oocyte quality [43]. Thus higher IL-6 levels associated with impaired fertility are probably secondary to the effect on the oocyte spindle. In addition, patients with Chlamydia-induced salpingitis and upper reproductive tract inflammation which are established etiologies of infertility, demonstrated higher IL-6 levels in the infiltrating lymphocytes in the acute phase of the disease [44]. IL-6 can also alter ciliary beat frequency in human fallopian tube owing to its presence in the inflammatory milieu of peritoneal fluid thus affecting ovum pick up and implantation [45]. These findings indicate that various sources of IL-6 may affect the mature oocyte spindle and chromosomal structure in states of inflammation.

In conclusion, the oocyte spindle apparatus is of vital importance in the successful outcome of the reproductive process. Oxidative states can compromise oocyte quality by affecting the meiotic spindle based on in vitro studies. IL-6, generated in the process of oxidative stress, not only regulates the inflammatory milieu and assists in maintenance of chronic inflammatory state but also directly affects the metaphase-II oocyte spindle and contributes to infertility. The future directions in prevention of oocyte quality damage may not only focus on antioxidants but also on inhibitors of cytokine cascades.

## Materials and Methods

### Materials

Metaphase-II mouse oocytes were obtained commercially (Embryotech Inc., MA) in cryopreserved straws. Recombinant mouse IL-6, anti-alfa tubulin antibody, fluorescein isothiocyanate (FITC) conjugate anti goat antibody, and propidium iodide were purchased from Sigma Aldrich (St. Louis, MO). Human tubular fluid (HTF) media was obtained from Vitrolife (San Diego, CA).

### Methods

The oocytes were transferred in phosphate buffer saline (Dulbecco's PBS) Sigma Aldrich, (St.Louis, MO) and washed to remove excess cryoprotectant for 3 minutes. This was followed by transferring the oocytes in HTF media and incubated at 37°C and 5% CO_2_ for 60 minutes for complete repolymerization. The oocytes were then screened for presence of polar body confirming their Metaphase-II stage. The oocytes were divided equally into unexposed controls and IL-6 treated groups (50,100 and 200 ng/mL). The end points of the experiments involved the assessment of microtubule and chromosomal alignment as previously described [26], [32]. Results obtained were compared in each experimental set between different groups using appropriate statistical tests.

### Immunostaining and fluorescence microscopy

Oocytes were fixed in a solution containing 2% formaldehyde, 0.2% Triton X-100 for 30 minutes. Fixed oocytes underwent indirect immunostaining using mouse anti-alfa tubulin antibody against the microtubules as the primary and FITC conjugate anti-goat antibody as the secondary antibody. The chromosomes were stained using propidium iodide. Stained oocytes were loaded into antifade agent (Biomedia, CA) on marked slides that have two etched rings. Scores were assigned 1 to 4 for both microtubule and chromosomal alterations based on previous published data [26]. Scores 1 and 2 were combined as “Good” score and 3 and 4 as “Poor” score. This was done since no statistical differences were found between scores 1 and 2 and similarly between 3 and 4. This also increased the power of the analysis. The alterations in the microtubules and chromosomes were compared with controls and scored by two different blinded observers (Figure 1) [26]. Images were obtained both utilizing immunofluorescent and confocal microscopy.

### Confocal Microscopy, assessment of Microtubules and chromosomal alignment

Confocal images were obtained utilizing a Zeiss LSM 510 META NLO (Zeiss LSM 510 META, Jena, Germany) microscope. Oocytes were localized using a 10× magnification lens and spindle alterations assessed using 100× oil immersion lens. The microtubules were stained fluorescent green, which was distinct from the red fluorescent staining of chromosomes. Individual treated and control oocytes in each experiment set were closely examined for spindle status. The categorization of oocytes based on MT and CH status was performed by two observers blinded to treatment group assignment, who used comprehensive evaluation of the individual optical sections and the 3-D reconstructed images (Figure 3A–D).

### Statistical Analysis

Data were analyzed using SPSS v.19.0 for Windows (IBM Corp.). Pearson Chi square was utilized for comparisons between control and induced groups. Comparisons among pairs of groups were analyzed with Fisher's Exact tests with correction for multiple comparisons were made using the Holm modified Bonferroni correction. Statistical significance was determined by a p<0.05.
