# Correction: IL-6 and Mouse Oocyte Spindle

**DOI:** 10.1371/annotation/947afede-1969-4061-a535-164655e9de72

**Published:** 2012-05-17

**Authors:** Jashoman Banerjee, Rakesh Sharma, Ashok Agarwal, Dhiman Maitra, Michael P. Diamond, Husam M. Abu-Soud

There was an error in Figure 3. The panels were labeled incorrectly. The correct Figure 3 can be viewed here: http://www.plosone.org/corrections/pone.0035535.gn003.cn.tif

